# Barriers to Interconception Care Delivery in Primary Care: Clinician, Staff, and Patient Perspectives

**DOI:** 10.1089/whr.2024.0110

**Published:** 2024-12-13

**Authors:** Caitlin Somerville, Hannah Shireman, Amanda Geppert, Ashley McHugh, Emily White VanGompel, Jane L. Holl, Debra B. Stulberg

**Affiliations:** ^1^ Pritzker School of Medicine, University of Chicago, Chicago, Illinois, USA.; ^2^ Department of Family Medicine, University of Chicago, Chicago, Illinois, USA.; ^3^ Center for Interdisciplinary Inquiry and Innovation in Sexual and Reproductive Health, Department of Obstetrics & Gynecology, University of Chicago, Chicago, Illinois, USA.; ^4^ Department of Family and Community Medicine, University of Illinois at Chicago, Chicago, Illinois, USA.; ^5^ Department of Neurology, University of Chicago, Chicago, Illinois, USA.

**Keywords:** interconception care, pregnancy, primary care, health care delivery, reproductive health care, qualitative research, human-centered design

## Abstract

**Purpose:**

Many reproductive age women, cared for routinely by primary care providers (PCPs), would benefit from interconception care, yet a minority of primary care visits include interconception care. This study assessed barriers to providing interconception care from the perspective of primary care clinicians, staff, and patients.

**Materials and Methods:**

Clinicians (*n* = 11), staff (*n* = 14), and patients eligible for interconception care (*n* = 6) from three primary care clinics in Chicago, Illinois participated in focus groups or interviews. Sessions with clinicians and staff elicited descriptions of their clinic’s current care delivery processes; sessions with patients focused on their experiences accessing care following pregnancy. Data were used to produce a process map and to identify barriers and facilitators to providing interconception care. Sessions were audio-recorded, transcribed, and thematically analyzed using Dedoose. Findings on barriers are presented here.

**Results:**

Processes for clinics to identify patients eligible for interconception care are lacking. PCPs do not routinely receive information about their patients’ prior pregnancies, and relevant information can be hard to access. While patients describe many care needs between pregnancies, they are unsure of where to turn for help: their PCP, obstetrical clinician, or other sources. Contributing organizational limitations involve clinic structure, appointment availability, resources, and insurance coverage.

**Conclusions:**

Multiple barriers in current primary care systems and processes contribute to poor interconception care delivery. These findings, given the known benefits of interconception care, can inform human-centered design to overcome barriers.

## Introduction

Interconception care is any service provided between pregnancies to improve health outcomes of a future pregnancy.^1^ In the United States, the average woman has at least three risk factors for experiencing future adverse pregnancy outcomes,^2^ and approximately a third of pregnancies occur after a short interpregnancy interval, defined as <18 months from delivery to subsequent conception.^3^ Short interpregnancy interval is associated with higher risk of preterm birth, and severe short interval (<6 months) is associated with increased maternal morbidity.^4^ Additionally, there is strong evidence that providing people with contraceptive care and other preventive reproductive health services leads to multiple health benefits, including reducing cancer and infection risk and preventing downstream pregnancy morbidity.^5^

Most reproductive age women have at least one physician visit per year, often with a primary care provider (PCP),^6,7^ offering opportunity to address interconception health. Yet, only 14% of outpatient visits include any sort of preconception or contraceptive care.^8^ US women living in poverty are disproportionately likely to receive routine reproductive care from PCPs rather than women’s health specialists such as obstetrician/gynecologists (Ob/Gyns).^7^ Low-income women face an elevated risk of adverse pregnancy outcomes,^9^ thus it is essential to maximize PCPs’ ability to deliver interconception care to reduce disparities and improve outcomes.

The purpose of this study was to gain clinician, staff, and patient perspectives on current systems and processes of primary care to understand missed opportunities and barriers to the provision of interconception care within primary care. The study team is interested in interconception care for all people who can become pregnant, regardless of gender identity; when citing research or quoting a study participant, we use the terminology (*e.g.,* “women of reproductive age,” “patients,” or “mothers”) used by the original publication or speaker.

## Materials and Methods

We collected qualitative data using virtual focus groups and interviews with primary care clinicians and staff, and separately, with patients eligible for interconception care. The University of Chicago Institutional Review Board approved this study.

### Recruitment sites and participants

We recruited participants from three diverse primary care practices in Chicago and the surrounding suburbs (Table 1). At each clinic, the research team identified clinician leaders who invited other clinicians and staff who interact with reproductive age patients to participate. The goal was to recruit at least one representative of each clinician type and relevant staff role. For patient recruitment, adults coming to the clinic for a health care visit were approached by a research team member to assess their study eligibility. Given the study’s focus on the interconception period, patients who had previously been pregnant (with no defined time for prior delivery) and were able to become pregnant again (*i.e.,* had not experienced tubal ligation, hysterectomy, or menopause) were eligible to participate. All study participants had to be at least 18 years of age or older. Clinic staff also provided information about the study to their patients who, if interested, could authorize the clinic to share their contact information with the study team, complete a screening form in University of Chicago’s Research Electronic Data Capture (REDCap), or reach out to the research team directly.

**Table 1.** Primary Care Clinic and Study Participant Characteristics

	Clinic A	Clinic B	Clinic C
Clinics			
Practice setting	Suburban	Urban	Urban
Teaching clinic	Yes	Yes	No
Federally qualified health center	No	No	Yes
Participants (*n* = 31)	12	12	7
Physicians^a^	2 (17%)	5 (42%)	2 (29%)
Advanced practice clinicians^b^	0 (0%)	0 (0%)	2 (29%)
Clinical or administrative staff^c^	8 (67%)	4 (33%)	2 (29%)
Patients	2 (17%)	3 (25%)	1 (14%)

^a^ Internists, family physicians.

^b^ Physician assistants, nurse practitioners.

^c^ Registered nurses, licensed practical nurses, medical assistants, patient support associates, social workers, practice managers.

### Data collection

**FIG. 1.**
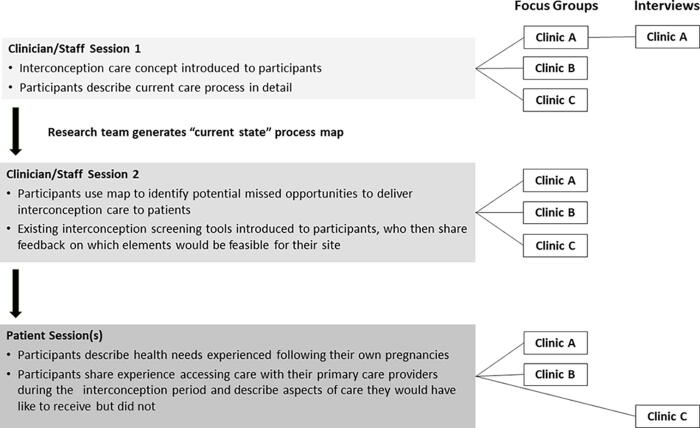
Flow chart of the data collection process.

Between April and October 2022, the research team held six focus groups with clinicians and staff and two with patients. For those interested and unable to attend the scheduled focus group time, we conducted individual interviews (Fig. 1). All sessions were conducted *via* Zoom (Zoom Video Communications, Inc),^10^ audio-recorded, and lasted approximately 60 minutes. To facilitate human-centered design data gathering techniques,^11,12^ we used Mural (Tactivos, Inc.),^13^ a digital workspace for visual collaboration. All participants provided informed consent. Sessions were moderated by research team members with collective experience in qualitative methods, process mapping, and human-centered design (A.G., J.L.H., D.B.S., E.W.V.G.).

At the end of each session, participants completed a brief demographic survey in REDCap and received a $30 e-gift card as compensation for their participation. Session recordings were transcribed verbatim and uploaded to Dedoose (version 9.0), a qualitative data management and analytic software.^14^

At the beginning of the first session, we defined interconception care to all participants as: health care after someone has given birth and before their next pregnancy. We explained that this includes contraceptive counseling to assist with pregnancy spacing, as well as assessing and addressing chronic conditions, preventive care needs, and risk factors that arose in prior pregnancies. For clinicians and staff, we also provided an illustrative case.

Clinician and staff sessions: Clinicians and staff were invited to participate in two consecutive sessions. In session 1, participants were asked to describe every step in their clinic’s current care delivery process. They described who interacted with patients and performed specific tasks, from scheduling and clinic check-in, through patient rooming, the clinical encounter, discharge from the clinic, and follow-up. Clinician and staff descriptions were used to create a “current state” process map of each clinic’s systems and processes of care, given differences in personnel, resources, and organizational characteristics of the three sites. The three maps were combined into a single map with annotation of alternative steps or processes and shared with participants in session 2. In session 2, participants validated the map and used it to identify potential missed opportunities, barriers, or facilitators to effectively deliver care. Finally, some existing preconception and interconception care tools were shown to clinicians and staff for consideration as potential solutions to the identified missed opportunities or barriers.

Patient sessions: Sessions with patients focused on their interconception care needs and experiences accessing care with their PCP following pregnancy. Patients were also asked to suggest ways primary care could better meet their needs in the interconception period.

### Thematic analysis

A codebook was developed by two team members (C.S., H.S.) who independently coded the first two transcripts and then met to reconcile their codes. The codebook was finalized through iterative discussion with the senior author (D.B.S.). Each transcript was then independently coded, and discrepancies were resolved until consensus. Excerpts of frequently used codes were reviewed and integrated into emergent themes.

In this article, we present the emergent themes regarding missed opportunities and barriers to the provision of interconception care in the setting of primary care. Facilitators and potential strategies will be reported separately.

## Results

A total of 31 clinicians, staff, and patients participated from three clinics (Table 2). All participants were asked to provide demographic information, however, only 64% chose to do so. Four overarching and interrelated themes emerged from our analysis.

**Table 2.** Participant Demographics

	Clinicians/staff (*N* = 25)	Patients (*N* = 6)
	*N* (%)	*N* (%)
Age		
20–29	4 (16%)	0 (0%)
30–39	6 (24%)	2 (33%)
40–49	6 (24%)	3 (50%)
50–59	2 (8%)	0 (0%)
Missing/Unknown^a^	7 (28%)	1 (17%)
Race		
American Indian or Alaska Native	1 (4%)	0 (0%)
Asian	2 (8%)	0 (0%)
Black or African American	3 (12%)	2 (33%)
White	8 (32%)	2 (33%)
More than one race	1 (4%)	0 (0%)
Other^b^	3 (12%)	1 (17%)
Missing/Unknown^a^	7 (28%)	1 (17%)
Ethnicity		
Hispanic or Latino	5 (20%)	2 (33%)
Not Hispanic or Latino	11 (44%)	3 (50%)
Missing/Unknown^a^	9 (36%)	1 (17%)
Gender		
Female	14 (56%)	6 (83%)
Male	4 (16%)	0 (0%)
Missing/Unknown^a^	7 (28%)	0 (0%)
Patient health insurance		
Personal/family’s job	N/A^c^	4 (66%)
Medicaid	N/A^c^	1 (17%)
Missing/Unknown^a^	N/A^c^	1 (17%)

^a^ Participant did not complete a demographic survey or respond to a specific question.

^b^ Participant self-reported “other,” including Arab, Middle Eastern, and Greek.

^c^ Insurance questions were not asked of clinicians and staff.

### Theme 1: Clinics lack reliable processes for identification of and outreach to patients eligible for interconception care

Identification: Across clinics, no established processes exist for PCPs to identify eligible patients who might benefit from interconception care. Inquiry into reproductive, contraceptive, and menstrual history during a clinic visit varied widely. One medical assistant (MA) said, “With pregnancies, we don’t really look too much into that.” Referring to a typical patient encounter, a clinician said:

“I don’t think there’s any specific questions about contraception or anything related to past pregnancy…That’s not part of our process.”

Another clinician highlighted that electronic health record (EHR) documentation of pregnancy and delivery care was not typically available to PCPs, even when the patient received care within the same health system (two of the participating clinics utilized the same EHR as their OB colleagues under the same health system, one clinic utilized an EHR not connected to providers outside their clinic). Across clinics, lack of access to relevant patient records left clinicians and staff without the information necessary to determine patient eligibility for interconception care: “I don’t think our system has any way of knowing that someone has been pregnant in the last X amount of time.” An MA added that even if the pertinent data were there, clinicians and staff may have difficulty finding it:

“If it’s a patient that has a lot of medical history and appointments, you’d probably have to do quite a bit of scrolling to even see that. So sometimes it’s not always …evident that they have had a child unless… the MAs look and see that.”

Outreach: Participants identified reliable outreach and scheduling as needs for effective interconception care delivery within primary care. One patient compared scheduling of newborn visits versus care for herself:

“They see you so often in the first week with the baby, like they scheduled those appointments…, but there was no one scheduling anything for [my] primary care.”

Patients expressed a desire for support from their primary care team, such as proactive engagement and general guidance, to help them care for themselves during this typically dynamic time:

“I don’t know who that conversation would be with, but I think it would have been nice if someone had reached out at like the six- or seven-month mark to say, hey let’s check in.”

Clinicians and staff discussed limited patient availability secondary to personal responsibilities, such as work and childcare, as a potential barrier. One patient support associate(PSA) shared:

“When moms do come in for their after visit from giving birth, they come in with their kids sometimes because they don’t have anybody to watch them.”

One clinician anticipated difficulty getting patients to prioritize an interconception care visit:

“Unless there’s an acute issue, they may just deprioritize it. A new mom, or even a mom for the second, third, or fourth time, is so busy that she puts her own needs at the top of the list, and it becomes the last thing she thinks about.”

While patients admitted that their own health was often not their greatest concern, they recognized the importance of taking care of themselves. One patient cited her children’s well-being as her primary motivation for addressing her own health concern: “I had problems with my thyroid… that really got me out the mold, like okay you got to get yourself together. You got kids you got to live for.” Patients proposed that clinics offer various options to increase accessibility, as another patient said:

“Even if it’s simple, it’s as simple as the video visit or if you have the option to come in in-person… Just let people know that it’s available and that someone is available. Cause you know, postpartum, a lot of time when you have a newborn your time schedule is not everybody else’s time schedule.”

### Theme 2: Gaps in care coordination between primary care and Ob/Gyn negatively impact patients’ experience receiving care

Participants across clinics acknowledged both PCP and Ob/Gyn clinicians as responsible for reproductive health care and pointed out that communication between the two is inconsistent. One clinician explained:

“It used to be automatic that we would get cc’ed charts on every specialist visit and… a lot of that was stopped so we now rarely get [charts]. The specialist has to be very intentional about sending it to us at this point.”

Patients shared their frustrations with the lack of coordination between their clinicians, as one said: “I don’t see why it’s my job to remember every visit I’ve ever had in my life. That information should be accessible somewhere to my current doctor.” Clinicians and staff thought that promotion of an intentional transition from Ob/Gyn to primary care could help bridge the gap after a delivery, and one MA suggested that Ob/Gyn start that conversation: “Like Ob/Gyn says, ‘Guess what, we surpassed this stage [pregnancy], here I hand off the baton…back to your primary care.’” Patients also thought that improvements in information gathering and sharing could help clinicians stay up to date on their experiences and alert them to potential concerns. One patient described her experience delivering in isolation during the COVID-19 pandemic and how she wished her clinicians were made aware of this and could prompt the conversation:

“Maybe there should be a big ol’ asterisk next to my name that says she had a freaking baby at lockdown, like let’s ask her about that… It would be really helpful to feel like my doctor is considering me as a person in my life around these medical events.”

### Theme 3: Patients lack knowledge about common interconception issues and which clinicians can address those concerns

Patients reported experiencing a wide range of health issues during the interconception period, including physical (*e.g.,* high blood pressure, hemorrhoids, hand pain, hair loss, urinary tract infection, pelvic floor pain, breast and lactation concerns, loss of libido) and emotional concerns (*e.g.,* anxiety, depression, family adjustment). Some patients reported that after receiving care from their Ob/Gyn during pregnancy, they felt uncertain about which clinician would be appropriate to contact postpartum. Patients indicated comfort with any expert addressing interconception care but shared that they had not thought of primary care as helpful or even appropriate to reach out to. One said, “I didn’t see my primary care doctor between pregnancies at all.” Instead, patients reported receiving advice from people in their lives. One patient described an interaction with her hair stylist, saying:

“I just started having all these little issues. They’re like, oh that’s from having a baby… I was like, really? I didn’t learn this in my parenting class. I don’t know, like who do I talk to now?”

One clinician pointed out that patients do not seem to fully understand their PCP’s role or scope of care: “Just being in family medicine, a lot of women aren’t aware that we deal with this stuff at all. I’ve had women who just say like, ‘Oh, I thought my OB needed to take care of this.’” PCPs reported that due to a lack of care coordination between clinicians, they rely heavily on patients to provide pertinent information, though it often falls to the PCP to elicit information: “When they come in it’s more like trying to weed out what it is we could address.” Clinicians and staff thought that patients could assume more responsibility in communicating their needs with clinicians, as a nurse highlighted:

“A lot of times people don’t necessarily think to talk about something… I don’t want to say [patients] should always be accountable for what they should be doing, but I mean there’s a piece of that as well.”

Patients reported feeling that they were expected to communicate their needs, but also preferred the clinician to initiate the discussion about “common changes in your body after pregnancy and what you should expect versus what you’re experiencing.” Patients differed in their level of comfort initiating these discussions with their clinicians, as one patient described the awkwardness she felt:

“I always feel really silly making an appointment and going in and them being like, ‘Okay everything’s fine’… So maybe if there were some kind of lower barrier to entry to kind of get those questions addressed, and then invite you in for an appointment if you need it.”

### Theme 4: Constraints posed by clinic structure, resources, appointment availability, and health insurance coverage impact feasibility of interconception care implementation

Clinicians and staff discussed practical constraints, including clinic structure and resources, appointment availability, and insurance coverage. After being introduced to existing interconception care tools, clinicians and staff discussed the feasibility of implementation at their sites. Participants from two clinics noted incongruence between their clinics’ current structures and the tools’ requirements:

“One of [the screening tools] needs the pre-visit planning that we don’t do. One of them needs the integrated kid and parent visit which very few of us do, so it might be harder to structure that.”

One of these clinics reported that they did not have the birth control options available to provide comprehensive contraceptive care to their patients and thus preferred to refer externally, such as to Planned Parenthood. A clinician said, “There are times that I do skip over because I’m like okay, there’s nothing else to do besides… birth control in terms of the pills… then we’re kind of stuck.” The other two clinics each had one clinician who could provide comprehensive reproductive health services. One clinician described an internal referral process for patients who wanted an intrauterine device (IUD) or contraceptive implant: “I’d say, ‘see my colleague…, she does IUDs and Nexplanons.’” These referral processes were not well known and lacked standardization, as one MA said:

“One of our PSAs asked me if my physician does IUDs and those kind of procedures, and I told her ‘no, only this doctor does,’ but it wasn’t clear to her how to go about scheduling the patient.”

One clinic emphasized time constraints during the visit as a concern, especially if patients come in for other reasons. A clinician said:

“We[‘ll] barely have the bandwidth to do this for physicals to be honest with you, so that’s going to be something we’ll have to figure out, but definitely [we] don’t have the bandwidth to do it for every visit.”

This clinician further discussed how adding any additional tasks during an encounter generally does not feel feasible: “It’s just another thing to perhaps add to a set of questions that occurs at the time of the visit, but that list can get very long.” Clinician fatigue came up as an issue that could cause interconception care to be overlooked even with an alert:

“You can get an **[EHR]** alert**…** for the physician to say, ‘hey, this patient has been pregnant, she’s of reproductive age, consider asking this question,’ and the physician will hit ignore and move on unfortunately.”

Clinicians and staff at this clinic also were not confident about having appointments scheduled solely for interconception care due to limited appointment availability: “We have long wait times as it is, so to add an additional visit is going to be really hard.” Patients across clinics reported trouble scheduling even general checkups and expressed frustration with unaddressed concerns and poor continuity of care. One patient shared her experience accessing care to address hand pain during the interconception period:

“I was like, is this a thing, I don’t know. My baby is really heavy, is that it? I can’t not pick him up. What do I do? And it took a few months but I finally, having a PPO, just looked somebody up that was available… and I was asked, like why aren’t you seeing your primary care? And it’s like Oh, cause she doesn’t have openings for six months, and my hand really hurts.”

Patients reported that accessibility issues were often exacerbated by insurance coverage limitations, for example, one patient said her insurance “wouldn’t cover any kind of actual lactation help, so that was a little frustrating.” Clinicians and staff across clinics also spoke about insurance barriers, such as a PSA who said:

“For insurance purposes when they go see the OB, that’s a specialist, so they get charged more than they would for a PCP… I’ve seen that happen often as well, where they’ve said, ‘Well I’ve seen my OB’ … Even though physicals are covered for a year with their insurance, they just figure they pay the specialist fees already.”

## Discussion

This study identified multiple barriers to the delivery of interconception care in current primary care systems and processes. Health system, individual clinic, and patient factors all contributed to missed opportunities for interconception care delivery in the participating clinics. These findings reinforce previous research on barriers to preconception care in primary care, which included limited clinician and patient knowledge, clinic and patient resource constraints, and confusion around primary care versus specialist roles.^15^ Patient preference for providers to initiate discussions and adapt to specific patient circumstances and preferences is also reinforced in other studies.^16–18^

Our interviews revealed specific coordination challenges in the delivery of interconception care, such as PCPs’ inability to identify eligible patients and patient barriers to seeking care while adjusting to life with a newborn. Innovative programs, combining mother–baby care together,^19^ or integrating interconception care into well child visits,^20–21^ offer promising strategies to address such barriers. However, newborn care is not universally colocated with adult primary care, and some participants in our study worried that dyadic parent–child care would not be feasible. Rather, they suggested that incorporation of interconception care solutions into existing primary care infrastructure would be more feasible.

Strengths of this study include a diverse group of clinicians, staff, and patients with varied reproductive health care experiences, yielding both breadth and depth of information on clinics’ current systems and processes of care. Broad, open-ended inquiry, with patients separate from clinicians and staff, provided participants a space to discuss what mattered most to them and share their experiences and reflections honestly. However, it is possible that patients and staff with particularly negative experiences may have been more motivated to participate, leading to some bias. While our results describe similar barriers and patient preferences to those reported from other primary care studies in difference settings,^15–18^ we drew a limited number of participants from three primary care clinics in the Chicago area and thus our study is not designed to be generalizable to all primary care settings. However, the credibility of our results is bolstered by our recruiting providers most likely to see reproductive-age patients at each clinic as well as clinical and administrative staff who are integral to a well-rounded understanding of the issue.

These findings illuminate important gaps in primary care for reproductive age patients. After giving birth, many patients have health needs and little sense of where to turn for care. Despite the existence of many preconception and interconception care tools for outpatient settings,^22^ primary care clinics have few, if any, systems or processes to identify and engage this patient population. Existing barriers to primary and reproductive care appear to be exacerbated during the interconception period. To mitigate reproductive health disparities and improve care for all, it is critical that primary care systems work to overcome these barriers. The findings from this study can inform future work, focused on applying human-centered design solutions to effectively implement interconception care delivery in primary care settings.

## Conclusions

Given the known benefits of interconception care, innovations are needed to overcome the multiple barriers in primary care to better support patients during the interconception period.

## Abbreviations Used

EHR: electronic health record
IUD: intrauterine device
MA: medical assistant
Ob/Gyn: obstetrician/gynecologist
PCP: primary care provider
PSA: patient support associate
